# The amount of late gadolinium enhancement outperforms current guideline-recommended criteria in the identification of patients with hypertrophic cardiomyopathy at risk of sudden cardiac death

**DOI:** 10.1186/s12968-019-0561-4

**Published:** 2019-08-15

**Authors:** Pedro Freitas, António Miguel Ferreira, Edmundo Arteaga-Fernández, Murrilo de Oliveira Antunes, João Mesquita, João Abecasis, Hugo Marques, Carla Saraiva, Daniel Nascimento Matos, Rita Rodrigues, Nuno Cardim, Charles Mady, Carlos Eduardo Rochitte

**Affiliations:** 1 Cardiovascular Magnetic Resonance and Computed Tomography Sector, Heart Institute (InCor), University of São Paulo Medical School, Avenida Dr. Enéas de Carvalho Aguiar, 44, Andar AB, Cerqueira César, São Paulo, SP 05403-000 Brazil; 2 Cardiology Department, Hospital de Santa Cruz, Centro Hospitalar de Lisboa Ocidental, EPE, Lisbon, Portugal; 3 Advanced Cardiovascular Imaging Department, Hospital da Luz, Lisbon, Portugal; 4 Laboratory of Genetics and Molecular Cardiology and Cardiomyopathies Unit, Heart Institute (InCor), University of São Paulo Medical School, São Paulo, Brazil; 5 Cardiovascular Department, Hospital dos Lusíadas, Lisbon, Portugal; 6 Radiology Department, Centro Hospitalar de Lisboa Ocidental, EPE, Lisbon, Portugal

**Keywords:** Hypertrophic cardiomyopathy, Risk stratification

## Abstract

**Background:**

Identifying the patients with hypertrophic cardiomyopathy (HCM) in whom the risk of sudden cardiac death (SCD) justifies the implantation of a cardioverter-defibrillator (ICD) in primary prevention remains challenging. Different risk stratification and criteria are used by the European and American guidelines in this setting. We sought to evaluate the role of cardiovascular magnetic resonance (CMR) late gadolinium enhancement (LGE) in improving these risk stratification strategies.

**Methods:**

We conducted a multicentric retrospective analysis of HCM patients who underwent CMR for diagnostic confirmation and/or risk stratification. Eligibility for ICD was assessed according to the HCM Risk-SCD score and the American College of Cardiology Foundation/American Heart Association (ACCF/AHA) algorithm. The amount of LGE was quantified (LGE%) and categorized as 0%, 0.1–10%, 10.1–19.9% and ≥ 20%. The primary endpoint was a composite of SCD, aborted SCD, sustained ventricular tachycardia (VT), or appropriate ICD discharge.

**Results:**

A total of 493 patients were available for analysis (58% male, median age 46 years). LGE was present in 79% of patients, with a median LGE% of 2.9% (IQR 0.4–8.4%). The concordance between risk assessment by the HCM Risk-SCD, ACCF/AHA and LGE was relatively weak. During a median follow-up of 3.4 years (IQR 1.5–6.8 years), 23 patients experienced an event (12 SCDs, 6 appropriate ICD discharges and 5 sustained VTs). The amount of LGE was the only independent predictor of outcome (adjusted HR: 1.08; 95% CI: 1.04–1.12; *p* <  0.001) after adjustment for the HCM Risk-SCD and ACCF/AHA criteria. The amount of LGE showed greater discriminative power (C-statistic 0.84; 95% CI: 0.76–0.91) than the ACCF/AHA (C-statistic 0.61; 95% CI: 0.49–0.72; *p* for comparison < 0.001) and the HCM Risk-SCD (C-statistic 0.68; 95% CI: 0.59–0.78; *p* for comparison = 0.006). LGE was able to increase the discriminative power of the ACCF/AHA and HCM Risk-SCD criteria, with net reclassification improvements of 0.36 (*p* = 0.021) and 0.43 (*p* = 0.011), respectively.

**Conclusions:**

The amount of LGE seems to outperform the HCM Risk-SCD score and the ACCF/AHA algorithm in the identification of HCM patients at increased risk of SCD and reclassifies a relevant proportion of patients.

**Electronic supplementary material:**

The online version of this article (10.1186/s12968-019-0561-4) contains supplementary material, which is available to authorized users.

## Introduction

Sudden cardiac death (SCD) is a rare but devastating outcome of hypertrophic cardiomyopathy (HCM). Implantable cardioverter defibrillators (ICD) have proved to be effective in the primary prevention of SCD among HCM patients but patient selection remains challenging, with different guidelines employing different tools to assess risk [1–3]. While the American College of Cardiology Foundation / American Heart Association (ACCF/AHA) guidelines rely on the presence of several classic risk factors, the European Society of Cardiology recommendations use a multiparametric score (HCM Risk-SCD) that estimates the 5-year risk of SCD [4]. Both these approaches have been clinically validated [5–8], but their discriminative power is suboptimal, and cases of disagreement between the two clinical criteria have the potential to create confusion among both clinicians and patients [9].

Late gadolinium enhancement (LGE) assessed by contrast-enhanced cardiovascular magnetic resonance (CMR) has the ability to non-invasively identify areas of myocardial fibrosis that are thought to constitute the substrate for life-threatening ventricular arrhythmias [10–17]. While myocardial LGE has emerged as a promising tool for SCD risk stratification in patients with HCM, its incremental utility and the way to integrate it in clinical decision making remain to be established. We hypothesized that the amount of LGE could outperform the current clinical criteria for ICD implantation in its ability to identify HCM patients at increased risk for SCD.

## Methods

### Study population

This study was a multicenter analysis of patients with HCM undergoing CMR for diagnostic confirmation and/or risk stratification at *Instituto do Coração* (InCor)*,* University of São Paulo (São Paulo, Brazil), *Hospital da Luz* (Lisboa, Portugal), *Hospital de Santa Cruz* (Lisboa, Portugal) and *Hospital dos Lusíadas* (Lisboa, Portugal). The initial study cohort consisted of 896 consecutive patients identified retrospectively in the databases maintained by the 4 laboratories. The Brazilian center included patients between 2003 and 2017 (*n* = 677) while the Portuguese centers included patients between 2009 and 2017 (*n* = 219). The diagnosis of HCM was made by experienced cardiologists using all available clinical data and based on typical features, with ventricular myocardial hypertrophy (left ventricular [LV] wall thickness ≥ 15 mm) occurring in the absence of any other disease responsible for hypertrophy [1]. Exclusion criteria comprised: 1) age < 16 years (*n* = 11); 2) CMR inconsistent with HCM or with an alternative diagnosis, such as athlete’s heart, Anderson-Fabry disease, cardiac amyloidosis or sarcoidosis (*n* = 53); 3) LV ejection fraction (LVEF) ≤ 35% by CMR (*n* = 5); 4) LGE pattern consistent with previous myocardial infarction (*n* = 3); 5) missing essential echocardiographic and/or 24 h Holter monitoring data (*n* = 313); 6) moderate or severe aortic/mitral disease (*n* = 13). Eighteen patients (2.0%) were lost to follow-up and were also excluded from the analysis. The study protocol was reviewed and approved by each institutions’ review board that waived the need for specific informed consent.

### Demographic, clinical history data and endpoint definitions

Data regarding clinical history and demographics were collected from patient chart review and electronic medical records. Familial history of SCD was defined as SCD in 1 or more first degree relatives under 40 years of age or SCD in a first degree relative with confirmed HCM at any age [4]. Information on genetic testing was available in 192 patients and was positive in 112 (58%).

Generally, all centers followed their patients at least once a year. Clinical status, annual echocardiography and Holter data were documented at the institution’s electronic medical records. The primary endpoint was a composite of SCD, aborted SCD, sustained ventricular tachycardia (VT), or appropriate ICD discharge. SCD was defined as witnessed sudden death with or without documented ventricular fibrillation (VF) or death within 1 h of new symptoms or nocturnal deaths with no antecedent history of worsening symptoms [18]. Event adjudication was performed by three Cardiologists who were blinded for the CMR data. Events were ascertained by reviewing electronic medical records including ICD electrograms. When death occurred outside of the hospital, the circumstances of death were determined by phone call interview with a family member. Any disagreement in event adjudication was discussed within this panel and solved by consensus.

### Echocardiographic, Holter monitoring and exercise test data

Echocardiography, 24 h Holter monitoring and exercise test data within a 6-month window in relation to the CMR study were collected. If no left ventricular outflow tract (LVOT) obstruction was found, we assumed a gradient of 3 mmHg since it is the minimum accepted by the HCM Risk-SCD. Left atrial diameter was determined by M-Mode or 2D in the parasternal long-axis plane [4]. Non-sustained VT (NSVT) was defined as ≥ 3 consecutive ventricular beats at a rate of ≥ 120 beats/minute and < 30 s in duration on 24 h Holter monitoring [4]. Abnormal blood pressure response in exercise testing was defined as either a failure to increase by ≥ 20 mmHg or a drop of ≥ 20 mmHg during effort [2].

### HCM risk-SCD and ACCF/AHA risk factors

The 5-year HCM Risk-SCD was calculated for each patient [4] and categorized into 3 risk strata [1]: low risk (< 4%, ICD generally not indicated); intermediate risk (4–5.9%, ICD may be considered) and; high risk (≥ 6%, ICD should be considered). The ACCF/AHA guidelines consider that ICD implantation is reasonable if patients present any of the following major risk factors: family history of SCD in first degree relatives, LV wall thickness ≥ 30 mm, or recent unexplained syncope. Also, ICD implantation can be useful (minor risk factors) if NSVT is registered in Holter monitoring or abnormal blood pressure response occurs with exercise testing.

### CMR data

All CMR scans were performed using a 1.5 T systems (Siemens Avanto® and Aera®, Siemens Healthineers, Erlangen, Germany; Toshiba Vantage Titan®, Toshiba Medical Systems, Tokyo, Japan; Phillips Achieva®, Phillips Healthcare, Best, the Netherlands; GE Signa CVi® General Electric Healthcare, Waukesha, Wisconsin, USA). Images were transferred to a core laboratory where an experienced cardiologist in CMR, blinded for clinical events, analyzed all CMR data using a dedicated software (Circle Cardiovascular Imaging® release 5.6.4, Calgary, Canada). LV volume, mass, and EF were measured by use of standard volumetric techniques. LV endocardial and epicardial borders on cine images were manually traced to define the myocardium, taking care to exclude papillary muscles and the intertrabecular blood pool. Maximal LV wall thickness was defined as the greatest dimension at any site within the LV myocardium.

In all centers, LGE images were acquired 10 min after the administration of 0.2 mmol/kg intravenous gadolinium chelate contrast agent with breath-hold 2-dimensional segmented inversion-recovery spoiled gradient echo sequence or phase-sensitive inversion-recovery sequences. Imaging was performed in short-axis views covering the LV from the mitral annular plane to the apex with 8 mm slice thickness and 2 mm gaps. The typical in-plane spatial resolution was 1.5 mm × 1.5 mm. Inversion time was optimized to null normal myocardial signal. The LV short-axis stack of LGE images was first assessed visually for the presence of LGE, followed by quantification when LGE was present. LGE was defined as areas of signal intensity ≥ 6 standard deviations from normal myocardium and was expressed as the percentage of total LV myocardial mass (LGE%). Any areas that were identified as LGE by the software, but deemed artifactual on visual analysis, were manually excluded. Finally, LGE% was categorized into four risk strata (0%, 0.1–10%; 10.1–19.9%; ≥ 20%) [10].

To evaluate interrater agreement, a second blinded cardiologist analyzed 50 random CMR studies. A strong correlation was achieved in the evaluation of LGE% between the two raters (Spearman’s Rho: 0.97; *p* <  0.001). A very good agreement was found between the two raters regarding LGE strata (weighted κ: 0.85; 95% CI: 0.73–0.96; *p* <  0.001).

### Statistical analysis

Categorical variables are presented as frequency and percentage, and continuous variables are presented as mean ± standard deviation (normal distribution) or median and interquartile range (non-normal distribution). Student’s t-test, Mann-Whitney U and Fisher’s exact test were used for comparison where appropriate. The amount of LGE in different risk categories was compared with a Kruskal-Wallis test.

Weighted κ was used to assess interrater agreement between the three classifications (HCM Risk-SCD, ACCF/AHA and LGE% strata). Quadratic weights were applied where the penalties for disagreement are milder for small disagreements but grow harsher as the disagreements become larger. The strength of agreement between each classification was considered poor (κ <  0.2), fair (κ = 0.21–0.4); moderate (κ = 0.41–0.6), good (κ = 0.61–0.8) or very good (κ = 0.81–1.0).

The effect of HCM Risk-SCD, ACCF/AHA and LGE on overall survival was assessed using Cox proportional hazards models. A sensitivity analysis was conducted with censoring at 5-years of follow-up since this is the time frame considered by the HCM Risk-SCD tool. Clinically relevant variables and/or variables with a *p*-value < 0.10 on individual analysis were included in multivariate models. The discriminative ability was assessed by calculation of the c index. For binary outcomes, the c index is identical to the area under the receiver operating characteristic curve [19]. Kaplan-Meier survival curves were plotted for each risk stratification tool. The log-rank test was used to assess for significant differences in time to endpoint between the risk strata. Net reclassification index (categorical NRI) was used to ascertain if LGE improves the risk stratification strategies of the American and European guidelines. Statistical significance was set at *p*-value < 0.05 (two-sided). All analyses were performed using SPSS® 25.0 and MedCalc® 9.3.8.0.

## Results

The baseline patient characteristics of the final population of 493 patients available for analysis are summarized in Table 1. A weak correlation was found between indexed LV mass and the amount of LGE (Spearman rho = 0.15, *p* <  0.001).

**Table 1:** Demographic and clinical characteristics

	Overall (*n* = 493)	No endpoint (*n* = 470)	Endpoint (*n* = 23)	*p*-value
Age (years)	46 (33–60)	47 (33–59)	37 (27–61)	0.129
Male sex, n (%)	285 (57.8%)	274 (58.3%)	11 (47.8%)	0.321
Clinical history				
Family history of SCD, n (%)	62 (12.6%)	59 (12.6%)	3 (13.0%)	0.945
Unexplained syncope, n (%)	64 (13.0%)	61 (13.0%)	3 (13.0%)	0.993
Known AF, n (%)	84 (17.0%)	75 (16.0%)	9 (39.1%)	0.004
Beta-blockers, n (%)	364 (73.8%)	346 (73.6%)	18 (78.3%)	0.621
Calcium channel blockers, n (%)	52 (10.5%)	49 (10.4%)	3 (13.0%)	0.690
ACEi/ARB, n (%)	169 (34.3%)	161 (34.3%)	8 (34.8%)	0.959
Holter monitoring data				
NSVT, n (%)	94 (19.1%)	86 (18.3%)	8 (34.8%)	0.049
Echocardiography data				
Left atrial size (mm)	43 (38–47)	42 (38–47)	47 (40–52)	0.025
MWT (mm)	19 (16–23)	19 (16–23)	20 (17–26)	0.213
MWT ≥ 30 mm, n (%)	35 (7.1%)	31 (6.6%)	4 (17.4%)	0.049
LVOTO (mm Hg)	6 (3–50)	6 (3–50)	10 (3–48)	0.472
LVOTO ≥ 30 mmHg, n (%)	174 (35.3%)	165 (35.1%)	9 (39.1%)	0.693
Exercise test data^a^				
Abnormal BP response, n (%)	29 (10.2%)	28 (10.3%)	1 (9.1%)	0.897
CMR data				
LVEF (%)	67 (61–70)	67 (61–70)	66 (55–70)	0.259
LVEF 35–50%, n (%)	16 (3.2%)	13 (2.8%)	3 (13.0%)	0.007
Indexed EDV (mL/m^2^)	73 (62–83)	73 (62–83)	76 (63–88)	0.410
Maximum LV thickness (mm)	21 (17–24)	21 (17–24)	23 (17–28)	0.677
LV mass index (g/m^2^)	92 (75–114)	92 (75–114)	90 (77–120)	0.600
LGE present, n (%)	391 (79.3%)	368 (78.3%)	23 (100%)	0.012
LGE (g)	5 (0.6–14.2)	4.6 (0.6–13.8)	26.3 (12.7–36.8)	< 0.001
LGE (%)	2.9 (0.4–8.4)	2.7 (0.3–7.7)	12.0 (9.3–24.3)	< 0.001

Continuous variables presented as mean ± standard deviation or median (25th – 75th percentiles) where appropriate

*ACEi/ARB* angiotensin converting enzyme inhibitor / angiotensin II receptor blocker, *AF* atrial fibrillation, *BP* blood pressure, *CMR* cardiovascular magnetic resonance, *EDV* end-diastolic volume, *ESV* end-systolic volume, *LGE* late gadolinium enhancement, *LV* left ventricle, *LVEF* left ventricular ejection fraction, *LVOTO* left ventricular outflow tract obstruction, *MWT* maximum LV wall thickness, *NSVT* non-sustained ventricular tachycardia

^a^from 283 exercise tests performed

### Agreement between risk stratification tools

According to the ACCF/AHA criteria, ICD would ‘not be recommended’ in 57%, ‘could be useful’ in 13.8%, and would be ‘reasonable’ in 29.2%. According to the HCM Risk-SCD criteria, ICD would not be ‘indicated’ in 73.4%, ‘could be considered’ in 13.4%, and ‘should be considered’ in 13.2%. LGE was present in 79.3% of patients, with a median LGE% of 2.9% (IQR 0.4–8.4%). Amongst patients with LGE, the distribution was the following: 0.1 to 10.0% of the LV mass (*n* = 285, 72.9%), 10.1 to 19.9% (*n* = 63, 16.1%), and ≥ 20% (*n* = 43, 11.0%).

**Fig. 1:**
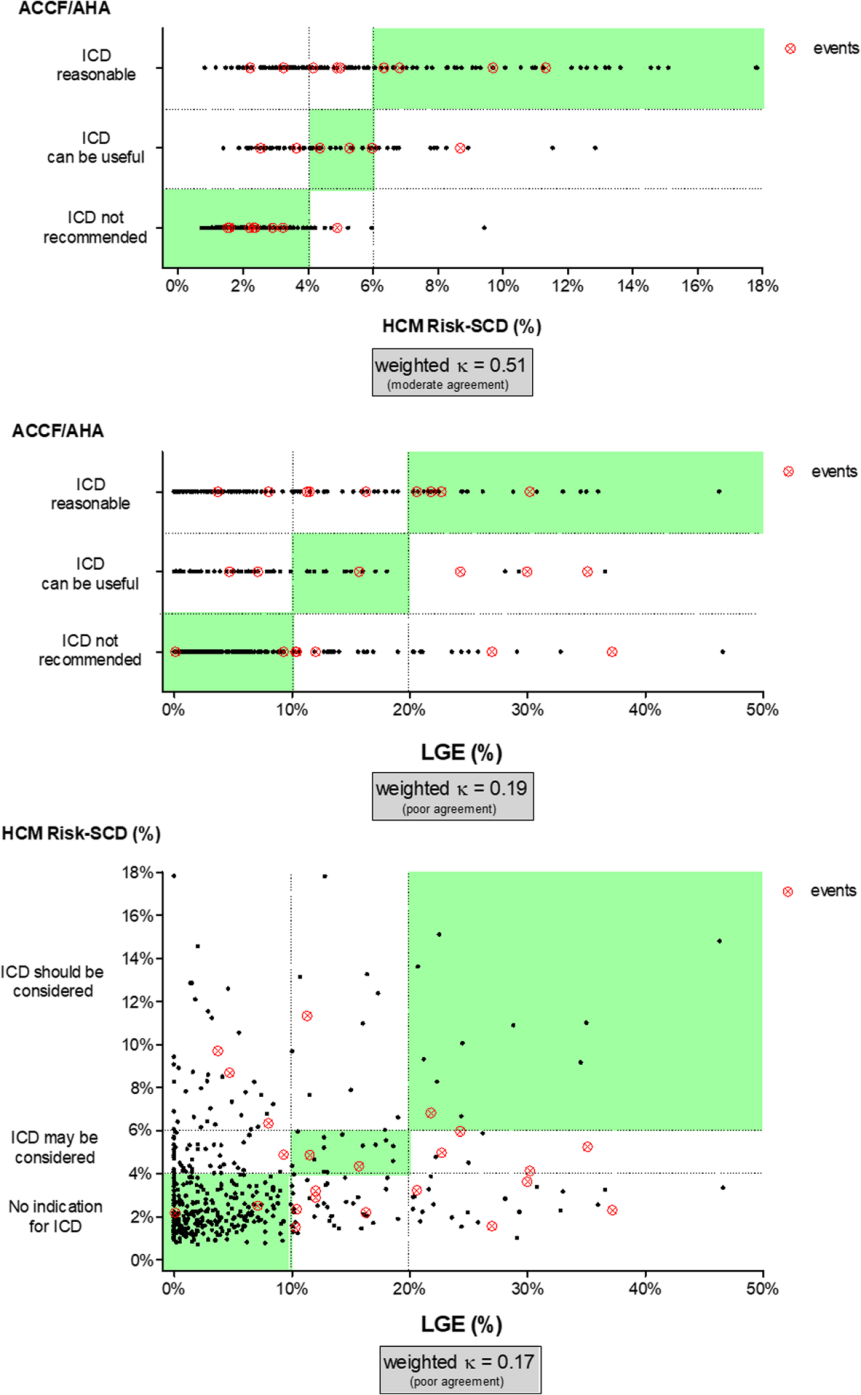
Agreement analysis between the ACCF/AHA algorithm, HCM Risk-SCD tool and LGE strata. Green background represents zones of concordance between classifications. ACCF/AHA – American College of Cardiology Foundation / American Heart Association; HCM-Risk SCD – hypertrophic cardiomyopathy risk sudden cardiac death; ICD – implantable cardioverter defibrillator; LGE – late gadolinium enhancement

The concordance between risk assessment by these methods was relatively weak (Fig. 1). Weighted κ analysis revealed moderate agreement between the HCM Risk-SCD and the ACCF/AHA classification [κ = 0.51 (95% CI: 0.44–0.58); *p* <  0.001]. Poor agreement was found between ACCF/AHA and LGE classifications [κ = 0.19 (95% CI: 0.11–0.27); *p* <  0.001] and between HCM Risk-SCD and LGE [κ = 0.17 (95% CI: 0.07–0.26); *p* <  0.001].

### Outcomes

The median total duration of follow-up was 3.4 years (IQR 1.5–6.8 years), during which a total of 23 events occurred (12 SCD, 6 appropriate ICD discharges and 5 sustained VT – Additional file 2: Table S1). An additional 4 cardiovascular deaths and 5 non-cardiac deaths were recorded. Throughout this period 42 ICDs were implanted for primary prevention. The distribution of events across each of the three studied classifications is presented in Table 2. A significant proportion of the patients who experienced an event were considered low-risk according to the clinical scores (35 and 48% for the ACCF/AHA and HCM Risk-SCD, respectively). Conversely, amongst those who were considered high-risk by the clinical scores, less than 10% experienced an event.

**Table 2:** Event distribution according to the studied classifications

	Overall (*n* = 493)	No endpoint (*n* = 470)	Endpoint (*n* = 23)	*p*-value
HCM Risk-SCD				0.018
Low risk, n (%)	362 (73.4%)	351 (74.7%)	11 (47.8%)	–
Intermediate risk, n (%)	66 (13.4%)	59 (12.6%)	7 (30.4%)	–
High risk, n (%)	65 (13.2%)	60 (12.8%)	5 (21.7%)	–
Risk at 5-years (%)	2.5 (1.7–4.1)	2.4 (1.7–4.0)	4.2 (2.4–6.0)	0.003
ACCF/AHA				0.075
ICD not recommended, n (%)	281 (57.0%)	273 (58.1%)	8 (34.8%)	–
ICD can be useful, n (%)	68 (13.8%)	62 (13.2%)	6 (26.1%)	–
ICD reasonable, n (%)	144 (29.2%)	135 (28.7%)	9 (39.1%)	–
LGE classification				< 0.001
0%, n (%)	102 (20.7%)	102 (21.7%)	0 (0%)	–
0.1–10.0%, n (%)	285 (57.8%)	279 (59.4%)	6 (26.1%)	–
10.1–19.9%, n (%)	63 (12.8%)	55 (11.7%)	8 (34.8%)	–
≥ 20%, n (%)	43 (8.7%)	34 (7.2%)	9 (39.1%)	–

Continuous variables presented as mean ± standard deviation or median (25th – 75th percentiles) where appropriate

*ACCF/AHA* American College of Cardiology Foundation / American Heart Association, *HCM Risk-SCD* hypertrophic cardiomyopathy sudden cardiac death risk tool, *LGE* late gadolinium enhancement

**Fig. 2:**
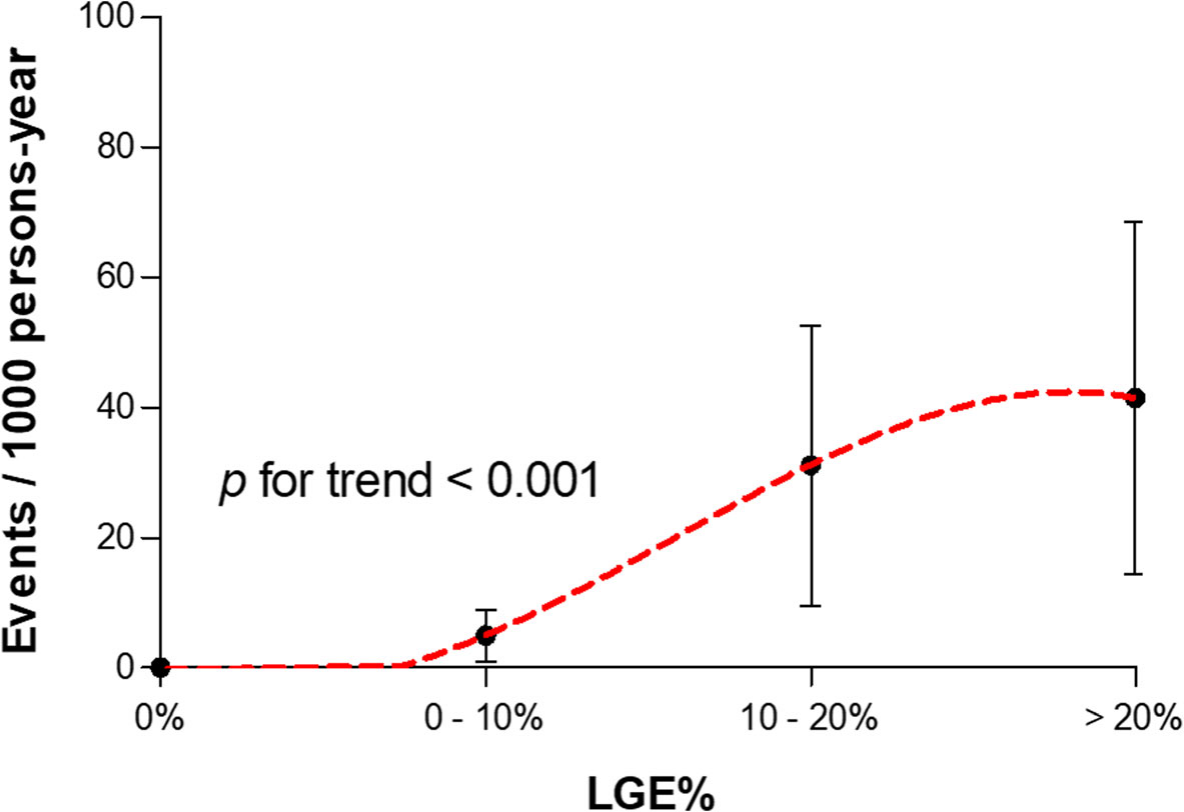
Unadjusted primary endpoint incidence per 1000 person-years according to the extent of LGE

The unadjusted primary endpoint incidence increased in direct relation to the extent of LGE (Fig. 2) ranging from 0 events per 1000 person-years (95% CI: 0–0.4) in patients without LGE to 41 events per 1000 person-years (95% CI: 14–68) in patients with LGE% ≥ 20%.

### Role of LGE% in risk stratification

**Fig. 3:**
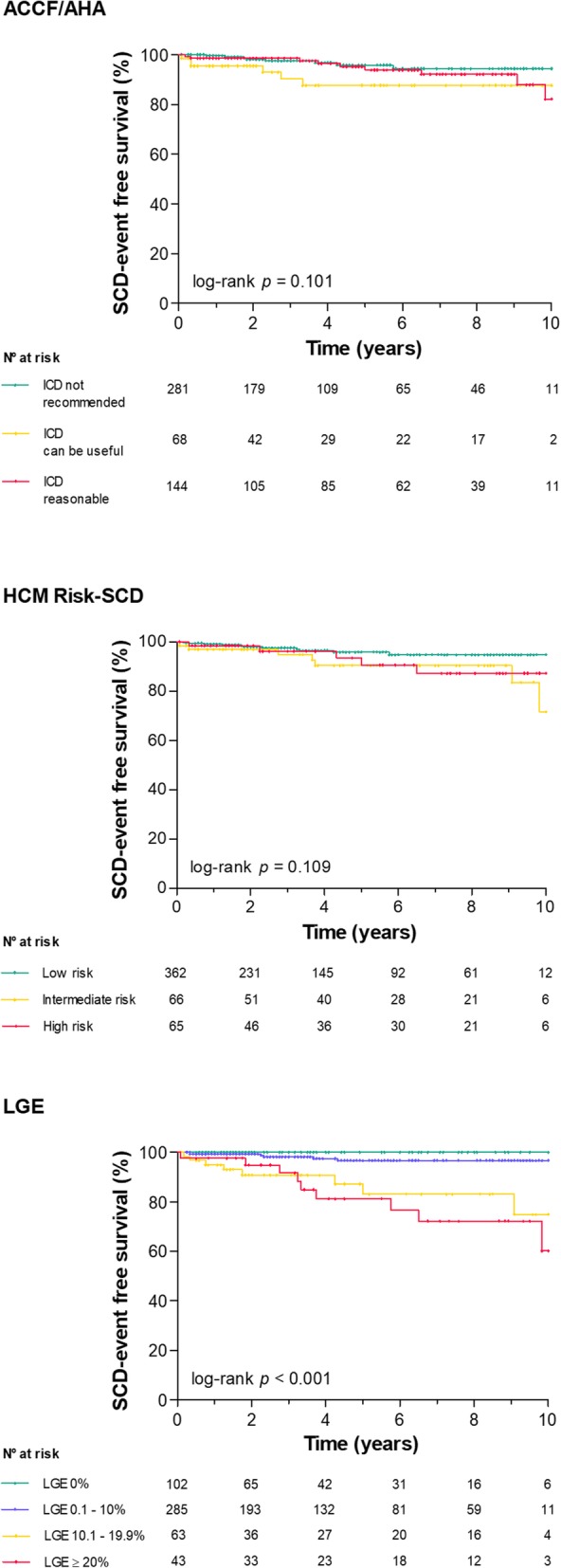
Survival analysis through Kaplan-Meier according to the ACCF/AHA, HCM Risk-SCD and LGE classifications

The amount of LGE showed greater discriminative power (C-statistic 0.84; 95% CI: 0.76–0.91) than the ACCF/AHA (C-statistic 0.61; 95% CI: 0.49–0.72; *p* for comparison < 0.001) and the HCM Risk-SCD strategies (C-statistic 0.68; 95% CI: 0.59–0.78; *p* for comparison = 0.006). Kaplan-Meier survival curves according to the pre-defined LGE% strata, HCM Risk-SCD strata and ACCF/AHA algorithm are depicted in Fig. 3. No statistically significant differences in SCD-event free survival were found between the risk strata of the HCM Risk-SCD and ACCF/AHA criteria (log-rank *p* = 0.109 and log-rank *p* = 0.101, respectively). In contrast, SCD-event free survival was significantly different between LGE% risk strata (log-rank *p* <  0.001).

Univariate analysis showed an association between SCD events and LGE%, LVEF, LA diameter, NSVT and known AF (Table 3). In multivariate analysis, the amount of LGE was the only independent predictor of outcome (adjusted HR: 1.08; 95% CI: 1.04–1.12; *p* <  0.001). To assess the incremental value of LGE%, two additional analyses were conducted, forcing the HCM Risk-SCD and ACCF/AHA into the models. Neither of these risk stratification tools modified the independent prognostic value of LGE%.

**Table 3:** Univariate and multivariate analysis using Cox regression hazards model

Univariate analysis			Multivariate analysis		
	HR (95% CI)	*p*-value	Model	HR (95% CI)	*p*-value
Age	0.996 (0.970–1.023)	0.783	Base model		
Male	0.638 (0.281–1.446)	0.282	LGE%	1.081 (1.044–1.120)	< 0.001
Known AF	3.299 (1.425–7.637)	0.005	Known AF	2.421 (0.903–6.490)	0.079
Unexplained syncope	0.926 (0.274–3.131)	0.901	LVEF	1.006 (0.951–1.064)	0.844
Family history of SCD	0.711 (0.236–2.682)	0.711	Left atrial size	1.036 (0.974–1.102)	0.259
NSVT	2.134 (0.903–5.042)	0.084	NSVT	1.121 (0.441–2.848)	0.810
Left atrial size	1.060 (1.016–1.105)	0.007	With HCM Risk-SCD		
LVOTO	1.004 (0.992–1.015)	0.516	HCM Risk-SCD	0.970 (0.866–1.086)	0.596
LVM > 30 mm	1.194 (0.516–2.763)	0.679	LGE%	1.087 (1.053–1.123)	< 0.001
LVMi	1.003 (0.995–1.011)	0.478	With ACCF/AHA		
LVEF	0.951 (0.903–1.002)	0.057	‘ICD not recommended’	reference	–
LGE%	1.083 (1.052–1.116)	< 0.001	‘ICD can be useful’	1.971 (0.664–5.847)	0.221
HCM Risk-SCD	1.034 (0.944–1.132)	0.476	‘ICD reasonable’	0.966 (0.354–2.636)	0.946
ACCF/AHA			LGE%	1.082 (1.049–1.117)	< 0.001
‘ICD not recommended’	reference	–			
‘ICD can be useful’	3.053 (1.058–8.809)	0.039			
‘ICD reasonable’	1.698 (0.652–4.422)	0.278			

*ACCF/AHA* American College of Cardiology Foundation / American Heart Association, *AF* atrial fibrillation, *HCM Risk-SCD* hypertrophic cardiomyopathy sudden cardiac death risk tool, *ICD* implantable cardiac defibrillator, *LGE* late gadolinium enhancement, *LVEF* left ventricular ejection fraction, *LVMi* left ventricular mass, indexed to body surface area, *LVOTO* left ventricular outflow tract obstruction, *NSVT* non-sustained ventricular tachycardia at Holter monitoring, *SCD* sudden cardiac death

The ability of LGE% to reclassify risk was further assessed by calculating the net reclassification index. The amount of LGE yielded an overall NRI of 0.43 (*p* = 0.011) when added to the HCM Risk-SCD, and an overall NRI of 0.36 (*p* = 0.021) when added to the ACCF/AHA algorithm – Table 4.

**Table 4:** Net reclassification improvements provided by LGE of the American and European risk strategies

		LGE		
		≤ 10%	10.1–19.9%	≥ 20%
ACCF/AHA algorithm				
No events	ICD not recommended	241	21	11
	ICD can be useful	47	12	3
	ICD reasonable	93	22	20
Events	ICD not recommended	2	4	2
	ICD can be useful	2	1	3
	ICD reasonable	2	3	4
Non-event NRI: 127/470 (0.27)				
Event NRI: 2/23 (0.09)				
Overall NRI: 0.36 (*p* = 0.021)				
HCM Risk-SCD				
No events	Low risk	298	32	21
	Intermediate risk	44	12	3
	High risk	39	11	10
Events	Low risk	2	5	4
	Intermediate risk	1	2	4
	High risk	3	1	1
Non-event NRI: 38/470 (0.08)				
Event NRI: 8/23 (0.35)				
Overall NRI: 0.43 (*p* = 0.011)				

*ACCF/AHA* American College of Cardiology Foundation / American Heart Association, *HCM Risk-SCD* hypertrophic cardiomyopathy risk sudden cardiac death, *ICD* implantable cardioverter defibrillator, *LGE* late gadolinium enhancement, *NRI* net reclassification index

### Sensitivity analysis

Since HCM Risk-SCD was derived to predict 5-year risk of SCD, we conducted a sensitivity analysis where follow-up was censored at 5 years. During this period, a total of 19 events were recorded (10 SCD, 5 appropriate ICD discharges and 4 sustained VT). Overall, findings remained similar (Additional file 1: Figure S1; and Additional file 3: Table S2, Additional file 4: Table S3, Additional file 5: Table S4, Additional file 6: Table S5).

## Discussion

Identifying the HCM patients who will most benefit from ICD implantation for the primary prevention of SCD remains challenging, owing largely to the heterogeneity of clinical and phenotypic expression and the relatively low event rate observed in this disease. Despite being clinically validated, the American and European HCM criteria for ICD implantation are limited by suboptimal discriminative power [5–8]. This notion is noticeable in our data where more than half of the patients who suffered an event were not considered ‘high risk’, and more than one third were even classified as ‘low risk’.

LGE has been shown to be an independent predictor of SCD in HCM patients but is yet to be fully integrated into clinical decision algorithms [12]. Albeit limited by the modest number of cases and events, our data suggest that the amount of LGE has greater prognostic value than the two clinical risk stratification tools and their individual components, and that using LGE for risk stratification would correctly reclassify a significant proportion of patients (net reclassification index ~ 0.40). These findings are in accordance with the evidence that myocardial fibrosis, as unveiled by LGE, is the pathophysiological substrate for malignant ventricular arrhythmias in HCM patients [20]. However, the apparent superiority of LGE over clinical risk scores does not mean that these should be abandoned or replaced by this imaging marker. Instead, we believe that an integration of clinical risk factors and the amount of LGE will probably yield the best results. In order to develop such a tool, a large and diverse population of HCM patients with comprehensive evaluation and long follow-up will be required [21].

To the best of our knowledge, our study is the first to perform a simultaneous comparison of the prognostic value of the amount of LGE with the two currently recommended risk stratification tools in an HCM population with a broad spectrum of SCD risk. With some exceptions, prior studies have shown consistent evidence of the prognostic capability of LGE in this setting [10–17]. In a non-selected population (*n* = 711, 22 SCD events) including patients with previous VT/VF, Ismail et al. found that LGE was not an independent predictor of SCD since it was superseded by LVEF [14]. Conversely, in a multicentric study by Chan et al. (*n* = 1293, 37 SCD events) the relative risk of SCD was higher with increasing amounts of LGE% [10]. More recently, a study in low/intermediate risk HCM patients with preserved LVEF (*n* = 1423, of whom 686 underwent myectomy; 60 SCD events) also showed an independent association between LGE and SCD events [11]. A meta-analysis of seven studies on the prognostic value of LGE showed that the risk of SCD is significantly associated not only with the presence of LGE, but also with the extent of LGE, even after adjustment for baseline characteristics (adjusted HR: 1.36/10% LGE; 95% CI: 1.10–1.69; *p* = 0.005) [12].

Several limitations of this study should be acknowledged. Patient inclusion was based on referral for CMR, and a significant proportion of patients had to be excluded due to missing echocardiography and/or Holter data. In addition, it is likely that some form of referral bias is present in our population that could have led to a higher baseline risk, especially when comparing with the studies by Chan et al. and Mentias et al. [10, 11]. Comparisons between the different risk models are also limited due to the small number of SCD events. Moreover, as rhythm documentation was not available for all SCD cases, some of these deaths may have been non-arrhythmic in nature. The thresholds for risk stratification according to the amount of LGE were taken from previous studies but are largely arbitrary and do not necessarily represent the best thresholds for considering ICD implantation. Further studies will also be needed to ascertain if the improvement in risk assessment justifies the costs of performing CMR and the availability issues it raises. Furthermore, it is possible that the amount of LGE may increase over time in some patients, which can influence risk assessment and event rates. Finally, we should emphasize that no risk score or imaging marker is a substitute for sound clinical reasoning and shared decision-making with well-informed patients.

## Conclusion

The amount of LGE outperforms the HCM Risk-SCD score and the ACCF/AHA algorithm in the identification of HCM patients at increased risk of SCD and is able to correctly reclassify a significant proportion of patients. This information may be considered in the clinical decision process.

## Additional file

Additional file 1: **Figure S1.** Survival analysis through Kaplan-Meier according to the ACCF/AHA, HCM Risk-SCD and LGE classifications with follow-up censored at 5-years. (TIF 285 kb)

Additional file 2: **Table S1.** Clinical and demographic characteristics of the 23 patients experiencing sudden cardiac death events. (DOCX 35 kb)

Additional file 3: **Table S2.** Demographic and clinical characteristics with follow-up time censored at 5-years. (DOC 75 kb)

Additional file 4: **Table S3.** Event distribution according to the studied classifications with follow-up time censored at 5-years. (DOC 49 kb)

Additional file 5: **Table S4.** Univariate and multivariate analysis using Cox regression hazards model with follow-up time censored at 5-years. (DOC 58 kb)

Additional file 6: **Table S5.** Net reclassification improvements provided by LGE of the American and European risk strategies with follow-up censored at 5-years. (DOC 50 kb)

## Data Availability

The datasets generated and/or analyzed during the current study are available from the corresponding author on reasonable request.

## Abbreviations

ACCF/AHA: American College of Cardiology Foundation / American Heart Association
AF: Atrial fibrillation
BP: Blood pressure
CMR: Cardiovascular magnetic resonance
EDV: End-diastolic volume
ESV: End-systolic volume
HCM: Hypertrophic cardiomyopathy
HR: Hazard ratio
ICD: Implantable cardioverter defibrillator
IQR: Interquartile range
LGE: Late gadolinium enhancement
LGE%: Percent late gadolinium enhancement
LV: Left ventricle/left ventricular
LVEF: LV ejection fraction
LVMi: Left ventricular mass indexed to body surface area
LVOT: Left ventricular outflow tract
MWT: Maximal wall thickness
NRI: Net reclassification index
NSTV: Non-sustained ventricular tachycardia
SCD: Sudden cardiac death
VF: Ventricular fibrillation
VT: Ventricular tachycardia
