# Supplementary figures and images for: The amount of late gadolinium enhancement outperforms current guideline-recommended criteria in the identification of patients with hypertrophic cardiomyopathy at risk of sudden cardiac death
Source: J Cardiovasc Magn Reson. 2019 Aug 15;21:50. doi: 10.1186/s12968-019-0561-4 (PMC6694533; doi:10.1186/s12968-019-0561-4)

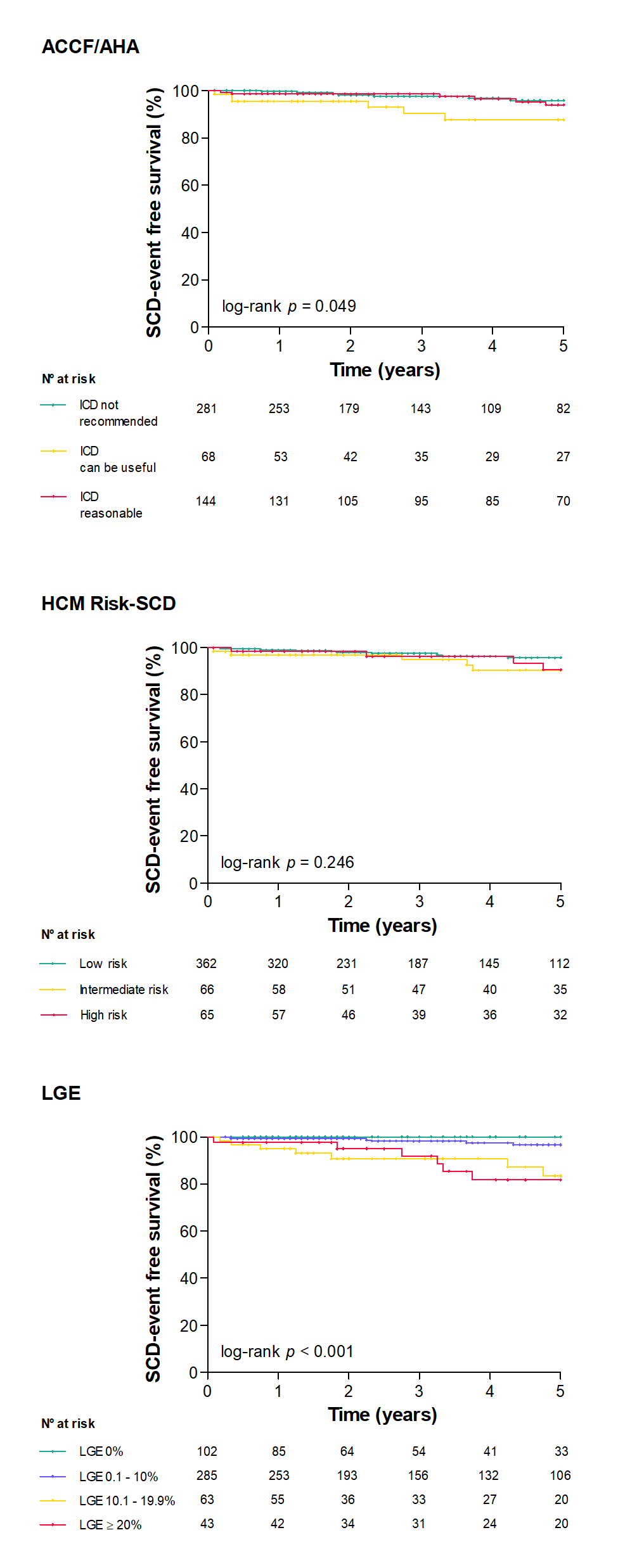

Supplement: Supplementary file 1 — Figure S1. Survival analysis through Kaplan-Meier according to the ACCF/AHA, HCM Risk-SCD and LGE classifications with follow-up censored at 5-years. (TIF 285 kb) [file 12968_2019_561_MOESM1_ESM.tif]
