# Supplementary material for: The amount of late gadolinium enhancement outperforms current guideline-recommended criteria in the identification of patients with hypertrophic cardiomyopathy at risk of sudden cardiac death
Source: J Cardiovasc Magn Reson. 2019 Aug 15;21:50. doi: 10.1186/s12968-019-0561-4 (PMC6694533; doi:10.1186/s12968-019-0561-4)
Supplement: Supplementary file 2 — Table S1. Clinical and demographic characteristics of the 23 patients experiencing sudden cardiac death events. (DOCX 35 kb) [file 12968_2019_561_MOESM2_ESM.docx]

**Additional file 2: Table S1** Clinical and demographic characteristics of the 23 patients experiencing sudden cardiac death events

|  |  |  | Transthoracic Echocardiogram | | | 24h Holter | Exercise test | CMR | | HCM Risk-SCD | | ACCF/AHA | | Event |
| --- | --- | --- | --- | --- | --- | --- | --- | --- | --- | --- | --- | --- | --- | --- |
| Patient | Age | Sex | MWT  (mm) | LA size  (mm) | LVOTO  (mmHg) | NSVT | Abnormal BP response | LVEF  (%) | LGE  (%) | 5-year risk (%) | Risk Strata | Risk factors*, n | ICD recommendation |  |
| #1 | 17 | M | 26 | 36 | 3 | no | no | 58 | 12.0 | 3.22 | low | 0 | ‘not recommended’ | SCD |
| #2 | 20 | M | 34 | 47 | 82 | no | no | 57 | 22.7 | 4.99 | intermediate | 1 | ‘reasonable’ | SCD |
| #3 | 25 | M | 39 | 42 | 3 | yes | no | 70 | 11.5 | 4.88 | intermediate | 2 | ‘reasonable’ | Sustained VT |
| #4 | 25 | M | 24 | 35 | 48 | no | no | 70 | 20.6 | 3.24 | low | 1 | ‘reasonable’ | SCD |
| #5 | 25 | M | 30 | 33 | 3 | yes | yes | 70 | 11.3 | 11.32 | high | 4 | ‘reasonable’ | Appropriate ICD therapy |
| #6 | 27 | M | 21 | 50 | 38 | no | NA | 70 | 8.0 | 6.33 | high | 1 | ‘reasonable’ | Sustained VT |
| #7 | 29 | F | 19 | 44 | 3 | no | NA | 51 | 24.3 | 5.96 | intermediate | 1 | ‘can be useful’ | SCD |
| #8 | 29 | M | 16 | 64 | 63 | no | NA | 70 | 3.7 | 9.70 | high | 1 | ‘reasonable’ | SCD |
| #9 | 31 | F | 15 | 48 | 10 | no | NA | 47 | 37.2 | 2.31 | low | 0 | ‘not recommended’ | SCD |
| #10 | 31 | F | 17 | 45 | 3 | no | no | 60 | 10.4 | 2.36 | low | 0 | ‘not recommended’ | SCD |
| #11 | 32 | F | 19 | 46 | 3 | no | NA | 55 | 30.2 | 4.15 | intermediate | 1 | ‘reasonable’ | SCD |
| #12 | 37 | F | 20 | 50 | 130 | no | NA | 51 | 9.3 | 4.89 | intermediate | 0 | ‘not recommended’ | Appropriate ICD therapy |
| #13 | 37 | F | 27 | 50 | 45 | yes | NA | 70 | 4.7 | 8.68 | high | 1 | ‘can be useful’ | Sustained VT |
| #14 | 42 | F | 15 | 35 | 17 | no | no | 70 | 16.3 | 2.21 | low | 1 | ‘reasonable’ | Appropriate ICD therapy |
| #15 | 45 | M | 22 | 59 | 10 | no | no | 70 | 21.8 | 6.81 | high | 1 | ‘reasonable’ | Appropriate ICD therapy |
| #16 | 55 | F | 16 | 47 | 85 | no | NA | 70 | 0.1 | 2.19 | low | 0 | ‘not recommended’ | Sustained VT |
| #17 | 55 | F | 18 | 38 | 36 | no | NA | 70 | 27.0 | 1.58 | low | 0 | ‘not recommended’ | SCD |
| #18 | 61 | M | 20 | 45 | 3 | yes | NA | 37 | 30.0 | 3.64 | low | 1 | ‘can be useful’ | Appropriate ICD therapy |
| #19 | 64 | F | 27 | 56 | 3 | yes | no | 67 | 35.1 | 5.27 | intermediate | 1 | ‘can be useful’ | Appropriate ICD therapy |
| #20 | 64 | M | 20 | 54 | 4 | yes | no | 41 | 15.7 | 4.36 | intermediate | 1 | ‘can be useful’ | Sustained VT |
| #21 | 65 | F | 24 | 38 | 20 | no | no | 58 | 10.3 | 1.52 | low | 0 | ‘not recommended’ | SCD |
| #22 | 76 | M | 15 | 52 | 3 | yes | NA | 66 | 7.1 | 2.53 | low | 1 | ‘can be useful’ | SCD |
| #23 | 77 | F | 20 | 59 | 120 | no | NA | 62 | 12.0 | 2.90 | low | 0 | ‘not recommended’ | SCD |

*ACCF/AHA* American College of Cardiology Foundation / American Heart Association, *AF* atrial fibrillation, *BP* blood pressure, *CMR* cardiac magnetic resonance, *HCM Risk-SCD* hypertrophic cardiomyopathy sudden cardiac death risk tool, *ICD* implantable cardiac defibrillator, *LA* left atrial, *LGE* late gadolinium enhancement, *LVEF* left ventricular ejection fraction, *LVMi* left ventricular mass indexed, *LVOTO* left ventricular outflow tract obstruction, *MWT* maximum LV wall thickness, *NSVT* non-sustained ventricular tachycardia, *SCD* sudden cardiac death, *VT* ventricular tachycardia.

* The major ACCF/AHA risk factors are (1) family history of SCD in first degree relatives, (2) LV wall thickness ≥ 30 mm, (3) recent unexplained syncope; and minor risk factors are (1) NSVT at Holter monitoring or (2) abnormal blood pressure response with exercise testing.
