# Supplementary material for: The amount of late gadolinium enhancement outperforms current guideline-recommended criteria in the identification of patients with hypertrophic cardiomyopathy at risk of sudden cardiac death
Source: J Cardiovasc Magn Reson. 2019 Aug 15;21:50. doi: 10.1186/s12968-019-0561-4 (PMC6694533; doi:10.1186/s12968-019-0561-4)
Supplement: Supplementary file 3 — Table S2. Demographic and clinical characteristics with follow-up time censored at 5-years. (DOC 75 kb) [file 12968_2019_561_MOESM3_ESM.doc]

**Additional file 3: Table S2** Demographic and clinical characteristics with follow-up time censored at 5-years

|  | **Overall**  **(*n* = 493)** | **No endpoint**  **(*n* = 474)** | **Endpoint**  **(*n* = 19)** | ***p*-value** |
| --- | --- | --- | --- | --- |
| Age (years) | 46 (33–60) | 46 (33–59) | 37 (29–64) | 0.483 |
| Male sex, n (%) | 285 (57.8%) | 197 (41.6%) | 11 (57.9%) | 0.157 |
| **Clinical history** |  |  |  |  |
| Family history of SCD, n (%) | 62 (12.6%) | 59 (12.4%) | 3 (15.8%) | 0.667 |
| Unexplained syncope, n (%) | 64 (13.0%) | 62 (13.1%) | 2 (10.5%) | 0.745 |
| Known AF, n (%) | 84 (17.0%) | 76 (16.0%) | 8 (42.1%) | 0.003 |
| Beta-blockers, n (%) | 364 (73.8%) | 349 (73.6%) | 15 (78.9%) | 0.605 |
| Calcium channel blockers, n (%) | 52 (10.5%) | 50 (10.5%) | 2 (10.5%) | 0.998 |
| ACEi/ARB, n (%) | 169 (34.3%) | 163 (34.4%) | 6 (31.6%) | 0.800 |
| **Holter monitoring data** |  |  |  |  |
| NSVT, n (%) | 94 (19.1%) | 87 (18.4%) | 7 (36.8%) | 0.044 |
| **Echocardiography data** |  |  |  |  |
| Left atrial size (mm) | 43 (38–47) | 42 (38–47) | 46 (38–52) | 0.097 |
| MWT (mm) | 19 (16–23) | 19 (16–23) | 20 (17–24) | 0.462 |
| MWT ≥ 30mm, n (%) | 35 (7.1%) | 33 (7.0%) | 2 (10.5%) | 0.553 |
| LVOTO (mm Hg) | 6 (3–50) | 6 (3–50) | 17 (3–48) | 0.554 |
| LVOTO ≥ 30 mm Hg | 174 (35.3%) | 166 (35.0%) | 8 (42.1%) | 0.526 |
| **Exercise test data^*^** |  |  |  |  |
| Abnormal BP response, n (%) | 29 (10.2%) | 28 (10.2%) | 1 (12.5%) | 0.831 |
| **CMR data** |  |  |  |  |
| LVEF (%) | 67 (61–70) | 67 (61–70) | 66 (55–70) | 0.308 |
| LVEF 35–50%, n (%) | 16 (3.2%) | 14 (2.9%) | 2 (10.5%) | 0.068 |
| Indexed EDV (mL/m^2^) | 73 (62–83) | 73 (62–83) | 76 (65–88) | 0.346 |
| Maximum LV thickness (mm) | 21 (17–24) | 21 (17–24) | 20 (17–27) | 0.851 |
| LV mass index (g/m^2^) | 92 (75–114) | 92 (75–114) | 90 (77–112) | 0.717 |
| LGE present, n (%) | 391 (79.3%) | 372 (78.5%) | 19 (100.0%) | 0.023 |
| LGE (g) | 5 (0.6–14.2) | 4.6 (0.6–13.9) | 20.7 (12.2–36.8) | < 0.001 |
| LGE (%) | 2.9 (0.4–8.4) | 2.8 (0.4–7.9) | 12.0 (8.0–24.3) | < 0.001 |

Continuous variables presented as mean ± standard deviation or median (25^th^ – 75^th^ percentiles) where appropriate.

*ACEi/ARB* angiotensin converting enzyme inhibitor / angiotensin II receptor blocker, *AF* atrial fibrillation, *BP* blood pressure, *CMR* cardiac magnetic resonance, *EDV* end-diastolic volume, *ESV* end-systolic volume, *LGE* late gadolinium enhancement, *LV* left ventricle, *LVEF* LV ejection fraction, *LVOTO* left ventricular outflow tract obstruction, *MWT* maximum LV wall thickness, *NSVT* non-sustained ventricular tachycardia.

^*^ from 283 exercise tests performed
