# Supplementary material for: The amount of late gadolinium enhancement outperforms current guideline-recommended criteria in the identification of patients with hypertrophic cardiomyopathy at risk of sudden cardiac death
Source: J Cardiovasc Magn Reson. 2019 Aug 15;21:50. doi: 10.1186/s12968-019-0561-4 (PMC6694533; doi:10.1186/s12968-019-0561-4)
Supplement: Supplementary file 4 — Table S3. Event distribution according to the studied classifications with follow-up time censored at 5-years. (DOC 49 kb) [file 12968_2019_561_MOESM4_ESM.doc]

**Additional file 4: Table S3** Event distribution according to the studied classifications with follow-up time censored at 5-years

|  | **Overall**  **(*n* = 493)** | **No endpoint**  **(*n* = 474)** | **Endpoint**  **(*n* = 19)** | ***p*-value** |
| --- | --- | --- | --- | --- |
| **HCM Risk-SCD** |  |  |  | 0.073 |
| Low risk, n (%) | 362 (73.4%) | 352 (74.3%) | 10 (52.6%) | – |
| Intermediate risk, n (%) | 66 (13.4%) | 61 (12.9%) | 5 (26.3%) | – |
| High risk, n (%) | 65 (13.2%) | 61 (12.9%) | 4 (21.1%) | – |
| Risk at 5-years (%) | 2.5 (1.7–4.1) | 2.5 (1.7–4.1) | 3.6 (2.4–6.0) | 0.015 |
| **ACCF/AHA** |  |  |  | 0.259 |
| ICD not recommended, n (%) | 281 (57.0%) | 274 (57.8%) | 7 (36.8%) | – |
| ICD can be useful, n (%) | 68 (13.8%) | 62 (13.1%) | 6 (31.6%) | – |
| ICD reasonable, n (%) | 144 (29.2%) | 138 (29.1%) | 6 (31.6%) | – |
| **LGE classification** |  |  |  | < 0.001 |
| 0%, n (%) | 102 (20.7%) | 102 (21.5%) | 0 (0%) | – |
| 0.1–10.0%, n (%) | 285 (57.8%) | 279 (58.9%) | 6 (31.6%) | – |
| 10.1–19.9%, n (%) | 63 (12.8%) | 56 (11.8%) | 7 (36.8%) | – |
| ≥ 20%, n (%) | 43 (8.7%) | 37 (7.8%) | 6 (31.6%) | – |

Continuous variables presented as mean ± standard deviation or median (25^th^ – 75^th^ percentiles) where appropriate.

*ACCF/AHA* American College of Cardiology Foundation / American Heart Association, *HCM Risk-SCD* hypertrophic cardiomyopathy sudden cardiac death risk tool, *LGE* late gadolinium enhancement.
