# Supplementary material for: The amount of late gadolinium enhancement outperforms current guideline-recommended criteria in the identification of patients with hypertrophic cardiomyopathy at risk of sudden cardiac death
Source: J Cardiovasc Magn Reson. 2019 Aug 15;21:50. doi: 10.1186/s12968-019-0561-4 (PMC6694533; doi:10.1186/s12968-019-0561-4)
Supplement: Supplementary file 5 — Table S4. Univariate and multivariate analysis using Cox regression hazards model with follow-up time censored at 5-years. (DOC 58 kb) [file 12968_2019_561_MOESM5_ESM.doc]

**Additional file 5: Table S4** Univariate and multivariate analysis using Cox regression hazards model with follow-up time censored at 5-years

| **Univariate analysis** | | |  | **Multivariate analysis** | | |
| --- | --- | --- | --- | --- | --- | --- |
|  | **HR (95% CI)** | ***p*-value** |  | **Model** | **HR (95% CI)** | ***p*-value** |
| **Age** | 1.002 (0.974–1.031) | 0.886 |  | **Base model** |  |  |
| **Male** | 0.509 (0.205–1.266) | 0.147 |  | LGE% | 1.069 (1.029–1.111) | < 0.001 |
| **Known AF** | 3.811 (1.531–9.487) | 0.004 |  | Known AF | 3.088 (1.072–8.894) | 0.037 |
| **Unexplained syncope** | 0.792 (0.183–3.429) | 0.755 |  | NSVT | 1.397 (0.515–3.793) | 0.511 |
| **Family history of SCD** | 1.059 (0.308–3.643) | 0.927 |  | LVEF | 0.999 (0.942–1.060) | 0.975 |
| **NSVT** | 2.459 (0.967–6.250) | 0.059 |  | Left atrial size | 1.019 (0.953–1.089) | 0.580 |
| **Left atrial size** | 1.052 (1.004–1.102) | 0.034 |  | **With HCM Risk-SCD** |  |  |
| **LVOTO** | 1.005 (0.993–1.017) | 0.429 |  | HCM Risk-SCD | 0.991 (0.878–1.118) | 0.887 |
| **LVM > 30 mm** | 1.309 (0.302–5.671) | 0.719 |  | LGE% | 1.076 (1.039–1.115) | < 0.001 |
| **LVMi** | 1.003 (0.993–1.012) | 0.567 |  | **With ACCF/AHA** |  |  |
| **LVEF** | 0.952 (0.900–1.007) | 0.085 |  | ‘ICD not recommended’ | reference | – |
| **LGE%** | 1.075 (1.040–1.111) | < 0.001 |  | ‘ICD can be useful’ | 2.391 (0.778–7.348) | 0.128 |
| **HCM Risk-SCD** | 1.046 (0.948–1.153) | 0.372 |  | ‘ICD reasonable’ | 0.859 (0.275–2.681) | 0.793 |
| **ACCF/AHA** |  |  |  | LGE% | 1.074 (1.037–1.112) | < 0.001 |
| **‘ICD not recommended’** | reference | – |  |  |  |  |
| **‘ICD can be useful’** | 3.531 (1.186–10.513) | 0.023 |  |  |  |  |
| **‘ICD reasonable’** | 1.390 (0.466–4.147) | 0.555 |  |  |  |  |

*ACCF/AHA* American College of Cardiology Foundation / American Heart Association, *AF* atrial fibrillation, *HCM Risk-SCD* hypertrophic cardiomyopathy sudden cardiac death risk tool, *ICD* implantable cardiac defibrillator, *LGE* late gadolinium enhancement, *LVEF* left ventricular ejection fraction, *LVMi* left ventricular mass, indexed, *LVOTO* left ventricular outflow tract obstruction, *NSVT* non-sustained ventricular tachycardia at Holter monitoring, *SCD* sudden cardiac death.
