# Supplementary material for: The amount of late gadolinium enhancement outperforms current guideline-recommended criteria in the identification of patients with hypertrophic cardiomyopathy at risk of sudden cardiac death
Source: J Cardiovasc Magn Reson. 2019 Aug 15;21:50. doi: 10.1186/s12968-019-0561-4 (PMC6694533; doi:10.1186/s12968-019-0561-4)
Supplement: Supplementary file 6 — Table S5. Net reclassification improvements provided by LGE of the American and European risk strategies with follow-up censored at 5-years. (DOC 50 kb) [file 12968_2019_561_MOESM6_ESM.doc]

**Additional file 6: Table S5** Net reclassification improvements provided by LGE of the American and European risk strategies with follow-up censored at 5-years

|  | | | **LGE** | | |
| --- | --- | --- | --- | --- | --- |
|  | | | **≤ 10%** | **10.1–19.9%** | **≥ 20%** |
| **ACCF/AHA algorithm** | | |  |  |  |
| **No events** | ICD not recommended | | 241 | 21 | 12 |
|  | ICD can be useful | | 47 | 12 | 3 |
|  | ICD reasonable | | 93 | 23 | 22 |
| **Events** | ICD not recommended | | 2 | 4 | 1 |
|  | ICD can be useful | | 2 | 1 | 3 |
|  | ICD reasonable | | 2 | 2 | 2 |
| Non-event NRI: 127/474 (0.27) | | | | | |
| Event NRI: 2/19 (0.11) | | | | | |
| Overall NRI: 0.38 (*p* = 0.036) | | | | | |
| **HCM Risk-SCD** | | |  |  |  |
| **No events** | | Low risk | 298 | 32 | 22 |
|  |  | Intermediate risk | 44 | 13 | 4 |
|  |  | High risk | 39 | 11 | 11 |
| **Events** | | Low risk | 2 | 5 | 3 |
|  |  | Intermediate risk | 1 | 1 | 3 |
|  |  | High risk | 3 | 1 | 0 |
| Non-event NRI: 36/474 (0.08) | | | | | |
| Event NRI: 6/19 (0.32) | | | | | |
| Overall NRI: 0.40 (*p* = 0.032) | | | | | |

*ACCF/AHA* American College of Cardiology Foundation / American Heart Association, *HCM Risk-SCD* hypertrophic cardiomyopathy risk sudden cardiac death, *ICD* implantable cardioverter defibrillator, *LGE* late gadolinium enhancement, *NRI* net reclassification index.
